# Phyto-Algal Consortia as a Complementary System for Wastewater Treatment and Biorefinery

**DOI:** 10.3390/plants14193069

**Published:** 2025-10-04

**Authors:** Huma Balouch, Assemgul K. Sadvakasova, Bekzhan D. Kossalbayev, Meruyert O. Bauenova, Dilnaz E. Zaletova, Sanat Kumarbekuly, Dariga K. Kirbayeva

**Affiliations:** 1 Department of Biotechnology, Faculty of Biology and Biotechnology, Al-Farabi Kazakh National University, Al-Farabi 71, Almaty 050038, Kazakhstan; huma.blch85@gmail.com (H.B.); asem182010@gmail.com (A.K.S.); bauyen.meruyert@gmail.com (M.O.B.); zaletova_dilnaz@mail.ru (D.E.Z.); kk.dariga@gmail.com (D.K.K.); 2 Tianjin Institute of Industrial Biotechnology, Chinese Academy of Sciences, No. 32, West 7th Road, Tianjin Airport Economic Area, Tianjin 300308, China; 3 Ecology Research Institute, Khoja Akhmet Yassawi International Kazakh-Turkish University, Turkistan 161200, Kazakhstan; 4 Department of Geography and Ecology, Abai Kazakh National Pedagogical University, Dostyk Avenue 13, Almaty 050010, Kazakhstan; 5 Department of Technosphere Safety and Analytical Chemistry, Altai State University, Krasnoarmeysky Avenue 90, Barnaul 656008, Russia; 6 Academic School General Education Disciplines, «Q» University, Bayzakov St. 125, Almaty 050026, Kazakhstan

**Keywords:** phytoremediation, phycoremediation, microalgae, aquatic macrophytes, consortium, wastewater, biomass valorization, circular bioeconomics

## Abstract

Pollution and freshwater scarcity, coupled with the energy sector’s continued dependence on fossil fuels, constitute a dual challenge to sustainable development. A promising response is biosystems that jointly address wastewater treatment and the production of renewable products. This review centers on a managed consortium of aquatic macrophytes and microalgae, in which the spatial architecture of plant communities, rhizosphere processes, and the photosynthetic activity of microalgae act in concert. This configuration simultaneously expands the spectrum of removable pollutants and yields biomass suitable for biorefinery, thereby linking remediation to the production of energy carriers and bioproducts within a circular bioeconomy. The scientific novelty lies in treating the integrated platform as a coherent technological unit, and in using the biomass “metabolic passport” to align cultivation conditions with optimal valorization trajectories. The work offers a practical framework for designing and scaling such consortia that can reduce the toxicological load on aquatic ecosystems, return macronutrients to circulation, and produce low-carbon energy carriers.

## 1. Introduction

Pollution of water resources driven by urbanization, industrialization, and climate change remains one of the most serious global threats to ecosystems, public health, and sustainable development [1,2]. Only about 0.3% of global water resources are accessible for practical use, which increases the vulnerability of aquatic systems [3]. Degradation is intensified by both natural factors (climate change, geochemical processes) and anthropogenic sources (agriculture, urbanization) [4]. At present, roughly 80% of the world’s wastewater is still discharged without adequate treatment, especially in developing countries [5]. Against this backdrop, environmentally sound and cost-effective approaches to pollutant removal, including biotechnological methods, are being actively explored [2]. At the same time, the global energy agenda is complicated by rising energy consumption and deep dependence on fossil fuels, which accelerates resource depletion and heightens climate risks [6]. The transition to renewable energy is a key condition for sustainable development and carbon-load reduction, whereas long-term reliance on fossil sources exacerbates water and air pollution and increases climate threats [7,8].

Conventional physicochemical treatment methods, including precipitation, adsorption, filtration, coagulation, flotation, and photocatalytic degradation, have proven efficacious; however, these methods frequently necessitate significant energy consumption, incur substantial financial costs, and often result in the generation of secondary waste [9,10]. In contrast, biological approaches rely on natural mechanisms of adsorption, absorption, assimilation, and biodegradation involving aquatic plants, microorganisms, and algae [11]. The resulting biomass opens up opportunities for producing biofuels, bioplastics, and fertilizers, linking water purification to the creation of renewable resources [12], and supporting the circular bioeconomy (CBE) [13]. These solutions align directly with the United Nations Sustainable Development Goals (SDGs)—clean water and sanitation, affordable and clean energy, health, and climate action and are viewed as the basis for multifunctional platforms for aquatic ecosystem restoration and bioenergy [14,15].

Among natural tools, aquatic macrophytes (AMs) play a special role. Plants such as *Eichhornia crassipes*, *Salvinia molesta*, *Lemna minor*, and *Pistia* stratiotes effectively remove a wide range of pollutants, including dyes and industrial impurities [16]. In some cases, removal of up to 99.9% of heavy metals (As, Cd, Cr, Hg, Pb), reduction in biochemical oxygen demand (BOD), and overall improvements in water quality are reported [17]. Efficiency is enhanced by oxygenation of the rhizosphere and stimulation of aerobic degradation of organics, and in constructed wetlands (CWs) through the action of microbial communities, adsorption, phytotransformation, and phytostabilization [18,19]. At the same time, AMs provide valuable biomass for bioenergy without competing for arable land and with lower resource demands [20]. For example, E. crassipes simultaneously reduces nitrogen, phosphorus, and organic loads and serves as feedstock for bioethanol and biohydrogen [21], while members of *Lemnaceae* (*Lemna*, *Wolffia*) are promising for bioethanol due to their high starch content (up to 70%) [22]. These advances are complemented by targeted adaptation of microalgae and macroalgae to remove specific pollutants [23].

Microalgae (MAs) are notable for their high photosynthetic efficiency, nutrient uptake, heavy metal binding, and organic matter degradation [11]. The species that attract the most attention include *Chlorella*, *Scenedesmus*, *Arthrospira*, *Chlamydomonas*, *Botryococcus*, *Ankistrodesmus*, and *Chlorococcum*, which combine rapid growth, lipid/protein accumulation, and a pronounced ability to absorb and biotransform pollutants. A significant advantage of many model microalgae is their genetic plasticity, which enables the use of modern gene-editing tools, for example, CRISPR-Cas systems, to precisely control of metabolic pathways, increase resistance to xenobiotics, and optimize metabolite composition. This, in turn, expands the range of biomass valorization applications [24]. Microalgae biomass can be obtained and converted into biofuels, such as biodiesel, bioethanol, and biohydrogen, making this technology economically attractive within the logic of a CBE [25]. An important advantage is the use of nutrients present in the wastewater itself, which reduces cultivation costs [26]. Integrating treatment with biomass production enables the simultaneous management of waste, generation of energy, and protection of the environment [27,28].

Synergistic integration of AMs and MAs enhances treatment performance, broadens the spectrum of removable pollutants, and creates direct channels for biomass valorization into bioenergy. AMs act as biofilters and rhizosphere oxygenators, whereas MAs function as powerful nutrient assimilators and biomass producers [29,30,31]. This integration yields multifunctional platforms that advance the CBE and reduce anthropogenic pressure on aquatic ecosystems.

Previous reviews of biotechnological wastewater treatment have focused primarily on single-organism platforms, such as microalgae monocultures or individual aquatic macrophytes. In contrast, this review analyses phyto-algal consortia, which are deliberately engineered synergies between aquatic macrophytes and microalgae, as an integrated system. The novel aspect is the cross-scale synthesis linking ecophysiology, metabolism, and process engineering to demonstrate how these consortia can simultaneously provide phytoremediation and biomass valorization under realistic operational and regulatory conditions. In particular, the review considers (1) the ecological and physiological basis of the synergy between AMs and MAs, (2) modern engineering solutions and technological advances for designing and scaling consortia, (3) opportunities for increasing the value of biomass within the CBE, and (4) key challenges and priority areas for future research. Thus, the work is not limited to stating recent results and gaps but offers an operational roadmap for the implementation of AM-MA systems: standard performance indicators, safe biomass application levels, and a focus on critical bottlenecks. The proposed perspective forms a practical agenda for the deployment of multifunctional AM-MA biosystems, capable of simultaneously enhancing environmental sustainability and resource and energy security in the 21st century.

## 2. Functional Ecologies of Phyto-Algal Consortia

In bioengineered systems, interspecific interactions extend beyond the confines of traditional ecology, forming synergistic groups capable of performing more complex tasks than monocultures and providing new functional capabilities [32]. In particular, the joint communities of AMs and MAs embody the principles of functional ecology, covering a wide range of relationships—from neutral coexistence to close mutually beneficial partnerships [33,34]. Thanks to the emergent properties of the consortium, the system demonstrates qualities that are not inherent in individual species, such as the ability to respond adaptively to external disturbances, which allows such communities to be considered a kind of “intelligent phytosystem” [35,36]. Understanding the “intelligence” of such systems rests on self-regulation via feedback [37]. Gas exchange, allelopathic regulation, microbial dynamics, and nutrient flux create a complex signaling network that optimizes consortium performance.

Nutrient cycling, pollutant degradation, and carbon sequestration are partitioned among different taxa within consortia, ensuring that system performance does not depend on any single species [38,39]. When designing such consortia, it is important to avoid one partner outcompeting the other: mechanisms of mutual checks and division of labor maintain interdependence between macrophytes and microalgae and prevent dominance by any single species. This increases functional redundancy and resilience: temporal and spatial complementarity among species enhances the stability of community functions under environmental fluctuations. AMs shape hydrodynamics, water–sediment interactions, and habitat structure, whereas MAs provide rapid nutrient uptake and efficient light use [40]. The combination of these functions imparts plasticity, resistance to fluctuations in irradiance and flow, and the capacity to detoxify xenobiotics through coordinated metabolic pathways [41]. During the process of consortium assembly, the expression of dominance is mitigated by the management of the resource environment (light, N:P stoichiometry, and flux partitioning) and by biochemical “check-points”. Macrophyte allelopathic signals and quorum-sensing regulation within epiphytic biofilms that modulate AM attachment and growth on AM surfaces stabilize cooperation through this feedback [42,43].

Acting as habitat engineers, AMs regulate light penetration, water chemistry, and microturbulence, creating niches for epiphytic and planktonic algae [44,45]. Their canopies buffer diel oxygen fluctuations: submerged surfaces generate diffusive boundary layers and local gradients of dissolved gases (O_2_, CO_2_, CH_4_, N_2_O), enabling reciprocal exchange of respiratory and photosynthetic products between partners [46]. Consequently, MAs are structurally dependent on AMs as sources of stable microgradients of light and oxygen and as substrates for epiphytic communities; in turn, MAs release dissolved organic carbon, which fuels the epiphytic microbiome and enhances oxidative processes at AM surfaces, improving water quality around leaves and roots. This bidirectional exchange of ecosystem services further counteracts shifts in dominance [47].

In the context of nutrient exchange, the process of AMs involves the absorption of inorganic nitrogen and phosphorus from sediments. This process has the effect of reducing competition with algae, and AMs exude organic compounds that stimulate MA growth [48,49]. MAs, in turn, can assimilate colloidal organic phosphorus and trace elements that are less accessible to AMs, sustaining coexistence even under resource fluctuations [50,51]. In mesocosms, mixed AM-MA communities often outperform monocultures in nitrogen and phosphorus removal due to spatiotemporal partitioning of nutrient uptake [52]. This supports the view that each partner exploits resources unavailable to the other and gains advantages only in the partner’s presence—complementarity that prevents a shift toward single-species dominance.

Buffering of the microscale environment arises from combined mechanisms: AM roots provide rhizofiltration and tissue-level metal sequestration, whereas MAs secrete extracellular polymeric substances (EPSs) that chelate and adsorb metal ions and organic xenobiotics; together, these processes lower bioavailability and toxicity and render the system more robust to chemical stressors, including fluctuations in pH, ionic strength/electrical conductivity, turbidity, and contaminant concentrations [53,54].

Beyond complementary resource use, interspecific chemical communication determines consortium structure and stability. AMs release allelochemicals—phenolics, terpenoids, and fatty acid derivatives that inhibit or selectively stimulate MAs, thereby suppressing harmful blooms [55,56]. Control is not unidirectional: MAs exude organic metabolites capable of reshaping the macrophyte root microbiome and modulating plant defenses and nutrient cycling [57].

Epiphytic bacteria on AM surfaces form biofilms that facilitate algal attachment and regulate allelopathic exchange [58]. In freshwater assemblages, such biofilms constitute dynamic communication networks in which bacterial quorum signals, algal metabolites, and macrophyte exometabolites intersect, governing colonization density, biofilm architecture, and interspecies cooperation [59]. These microbiome-mediated interactions are pivotal for maintaining system functionality [60]. Practically, bacterial communities should be treated as a manageable resource: MA-associated growth-promoting bacteria supply the consortium with vitamins (including B_12_), siderophores, and phytohormones, promote flocculation/sedimentation of biomass, and thereby facilitate harvesting of mixed AM-MA biomass. Phytohormones, most notably indole-3-acetic acid (IAA), are synthesised by bacteria and mycorrhizae. These compounds function as interkingdom signals, modulating mycorrhizal growth, morphogenesis, and metabolism. Moreover, they can exert indirect effects on arbuscular mycorrhizal physiology through the microbiome. The following essay will provide a comprehensive overview of the relevant literature on the subject. The administration of low-dose IAA and/or selection of bacterial IAA producers can be used to fine-tune consortium balance, complementing light and nutrient regimes [61,62].

The evolutionary history of AM-MA interactions highlights the significance of coevolution in ensuring consortium stability [63]. Contemporary engineering strategies are characterized by the deliberate implementation of analogous principles, such as function loss or partitioning, and the establishment of controlled interdependencies, in the selection of communities exhibiting desirable cooperative traits [64,65].

At the same time, there are no direct, well-substantiated cases of horizontal gene transfer (HGT) specifically between modern MAs and higher aquatic plants; documented examples pertain primarily to bacterial–algal and bacterial–plant lineages. Hence, the advantages of the consortium rest largely on metabolic cooperation and microbial mediation rather than gene exchange between MAs and AMs [66,67].

The transition-from-coexistence-to-synergy framework recognizes that AM-MA links are active, dynamic, and amenable to bioengineering. It is noteworthy that the model green alga *Chlamydomonas reinhardtii* now supports DNA-free CRISPR/Cas ribonucleoprotein (RNP) workflows, including HDR insertions, precise base substitutions, and endogenous tagging. These workflows exhibit high reproducibility and no marker integration, thereby opening up avenues for strains with tailored metabolic/sorptive traits [68,69,70,71].

In natural ecosystems, coexistence emerges from trade-offs between competition and mutualism, with species balancing resource competition against complementary benefits [72,73]. In engineered systems, deliberate design can move interactions beyond neutral coexistence toward genuine synergy. This transition requires control over environmental factors: surfaces for colonization, initial microbial densities, and regime parameters (light, nutrient ratios, hydrodynamics). Spatiotemporal heterogeneity, once viewed as destabilizing, can be leveraged to strengthen niche specialization and cooperative functions [74,75]. Design of AM-MA consortia demonstrates that intentional ecological cooperation can yield systems with exceptional robustness and efficiency. By integrating multilayered interactions, allelopathic regulation, microbiome mediation, and functional complementarity, these communities transcend natural symbioses to become truly synergistic engineered phytosystems. Modern omics and ecological modeling provide mechanistic insight into structure–function relationships under environmental heterogeneity and reveal evolutionary innovations [76,77]. Building on this foundation, synthetic-ecology paradigms enable the purposeful assembly of consortia with high phytoremediation efficacy and bioenergy productivity [78]. The shift from “symbiosis” to “synergy” highlights the transformative potential of ecological cooperation: rationally designed AM-MA systems can simultaneously support biodiversity, tolerance to toxicants, enhanced nutrient capture, and maximal biomass productivity.

## 3. The Metabolic Fates of Phyto-Algal Consortia Biomass

### 3.1. Biotransformation of Pollutants in Hybrid Phyto-Algal Systems

In hybrid AM-MA systems, the fate of pollutants and the resulting biomass is determined not by a simple sum of plant and algal mechanisms but by an integrated transition from primary sorption to intracellular processing and detoxification. This linkage of processes shapes the biomass profile and thus its suitability for subsequent valorization into energy carriers [79]. The sequential phases of biological pollutant removal observed in hybrid systems are analytically best distinguished as surface sorption, intracellular detoxification, and adaptive metabolic reprogramming. Over time these phases are interdependent and partially overlapping, and together they determine the suitability of biomass for valorization [79,80].

The first, surface phase is characterized by rapid, energy-independent binding of metal cations and polar xenobiotics at the microalgal cell surface. Cell walls and the exopolysaccharide matrix (carboxyl, hydroxyl, and amine groups) provide electrostatic attraction, complexation, and ion exchange, forming a surface barrier that retains part of the toxicants and limits their diffusion into the cell [81,82,83]. In the second, intracellular phase, part of the bound pollutant is transferred into the cytosol and vacuoles, and universal detoxification mechanisms are activated: enhanced chelation of metal ions by glutathione, phytochelatins, and metallothioneins, a reduction in free ion activity and reactive toxicity, and engagement of antioxidant circuits to constrain oxidative stress [84,85]. The third, adaptive–metabolic phase is expressed in a reconfiguration of microalgal biosynthesis and energy balance: the proportions of carbohydrates, proteins, lipids, and pigments shift, which affects photosynthetic efficiency and directly influences the potential fuel value of the biomass [86,87,88,89].

Experimental observations confirm the regulatory role of environmental factors. Acclimatization of *Desmodesmus* sp. MAS1 and *Heterochlorella* sp. MAS3 to pH 3.5 (relative to 6.7) under exposure to Cu, Fe, Mn, and Zn was accompanied by an increase in the relative fractions of proteins and lipids in both strains, while carbohydrates decreased in MAS1 and increased markedly in MAS3. Proper pH tuning mitigated toxicity by resetting metabolism [90]. In another study, Cr (0–500 µg/L) in *Mucidosphaerium pulchellum* and *Micractinium pusillum* caused a strong decline in Fv/Fm, together with an increase in intracellular lipids and a shift in fatty-acid profiles, a dual effect of reduced primary productivity with enrichment of the biomass in fuel-relevant components [91]. When interpreting results, it is important to account for the physiological duality of metal roles: at low concentrations, they act as structural elements of electron-transport and photolytic centers and as enzyme cofactors; in excess (including nonessential Hg, As, Cd, Pb, Cr), they disrupt photosynthesis, cell division, and enzymatic activity and induce morphological changes [92].

Macrophytes complement these mechanisms through well-developed root systems and rhizofiltration. Uptake of heavy metals and organic xenobiotics followed by sequestration in roots or translocation to aboveground tissues alters the distribution of toxicants in plant biomass and must be considered when planning subsequent use of the material [93,94,95,96]. Thus, a staged model for microalgae and macrophytes provides a clear interpretation of observed effects. Surface sorption limits pollutant entry, intracellular detoxification reduces reactive hazard, and adaptive metabolic reprogramming defines the qualitative composition of the biomass and its energy potential.

A comprehensive structured literature search was conducted in the Scopus, Web of Science, and PubMed databases for the period of January 2017 to August 2025. This was supplemented by a targeted “snowball” method using Google Scholar. The search queries employed in this study encompassed a combination of controlled terms and free keywords, with a focus on AMs, MAs, wastewater, and consortia. Two complementary trends can be identified in the extant literature: firstly, studies that examine microalgae separately [97,98,99,100,101,102,103] and macrophytes separately [104,105,106,107,108,109,110], and secondly, works that analyze their cooperation. As illustrated in Table 1, the extant empirical data on AM-MA consortia in real and model aquatic matrices has been collated and verified [111,112,113,114,115,116,117].

The data show that in hybrid AM-MA systems, the fate of pollutants and the properties of the biomass are governed by a controlled sequence of sorption, intracellular detoxification, and metabolic reprogramming. Targeted tuning of environmental conditions, strain selection, and post-harvest decontamination protocols ensure a reproducible feedstock quality and stabilize fuel yields, thereby converting contaminant-laden biomass into a safe, controllable resource for renewable energy.

### 3.2. Integrated Platform of Phytoremediation and Biorefinery Within a Circular Bioeconomy Framework

The phytoremediation concept, coupled with biorefinery complexes, treats the biomass generated during water treatment as a central resource of the CBE. The treatment stage, in which nutrients and pollutants are removed, is technologically coupled to subsequent processing of the resulting feedstock into energy carriers and bioproducts; in other words, the biomass is designed from the outset for downstream conversion rather than arising incidentally as a by-product [5,118]. In aquatic environments rich in nitrates, phosphates, and metals, AMs and MAs simultaneously extract undesirable components from the water and accumulate biopolymers—lipids, carbohydrates, and proteins—creating a favorable starting matrix for biotechnological and thermochemical processing [83,119,120,121,122]. An important feature of this feedstock is the plasticity of its composition, which reflects cultivation conditions, trophic status, and stress factors. When grown on wastewater under nitrogen limitation, MA–bacteria consortia, including communities dominated by *Chlorella* and/or *Tetradesmus*, often display rapid growth alongside a shift toward a higher carbohydrate content and a moderate lipid fraction, with a relatively smaller protein share; protein and carbohydrate fractions change in ways that are compensatory, while lipids act as an “energy bank” [119]. Metals and other stress factors can induce lipogenesis and modify the fatty-acid profile, improving the suitability of biomass for biodiesel, although this simultaneously requires control of co-pollutants in product streams [86]. In practical terms, controlled cultivation, the nitrogen regime, the salt and trace-element profile, and calibrated stress enable targeted shaping of the biomass “metabolic passport” for the intended valorization pathway: the protein fraction for biofertilizers/soil conditioners (where permitted and after meeting pollutant and pathogen thresholds), the carbohydrate fraction for alcoholic fermentation and biobutanol production, and the lipid fraction for transesterification into fatty acid methyl esters (FAMEs) [123,124].

Heavy metals (HMs) represent ubiquitous inorganic contaminants that exert profound ecological and toxicological impacts on humans and animals, whereas biomass retrieved from polluted matrices frequently accumulates these elements together with persistent organic xenobiotics, thereby posing risks of trophic transfer and constraining its suitability for food-related applications [125]. In MAs, metal tolerance is ensured by a sequence of extracellular and intracellular mechanisms, including biosorption, compartmentalization, synthesis of phytochelatins and metallothioneins, and efflux transport, which mitigate acute toxicity but do not remove metals from the biomass per se [126]. In such cases, energy and material routes take priority—fermentation, pyrolysis, and hydrothermal carbonization—where contaminants are managed by process controls, including immobilization and stream separation [122,127]. For thermochemical processes, it has been shown that thermodynamically volatile metals (Hg, Cd, As, Pb) partly decline, while thermally stable metals (Cr, Mn, Ni) concentrate in the solid phase; with appropriate operating conditions, low leachability and a high degree of immobilization in biochar can be achieved [128]. For AM biomass, composting and vermicomposting are environmentally sound complements that convert mobile metal forms into more stable oxidizable and residual fractions, reducing bioavailability [129,130,131].

**Figure 1:**
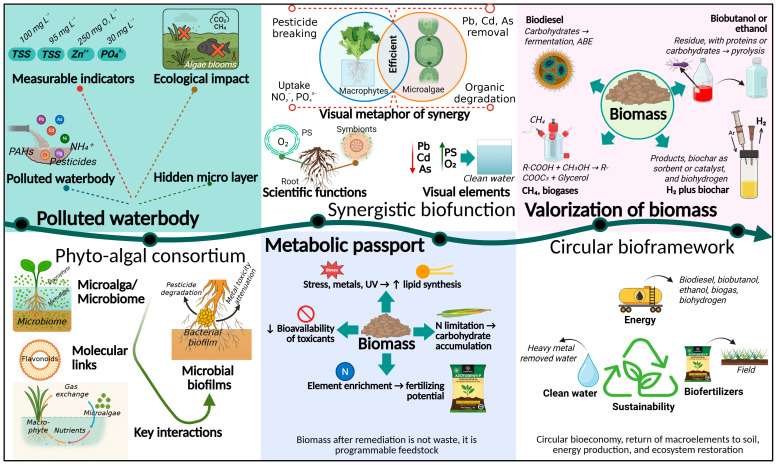
Transition from polluted water bodies to synergistic bioremediation and biomass valorization in the circular bioeconomy. Created with BioRender.com (agreement number: KX28NUTXC7). Available online: https://BioRender.com/r75dsdl (accessed on 26 August 2025).

The resulting “metabolic passport” defines suitable valorization routes. Figure 1 presents the integrated logic of the transition from a polluted water body to the formation of AM-MA consortia, followed by programmed shifts in protein, lipid, and carbohydrate ratios under nitrogen regimes and stressors, and then to a technological fork of biomass-processing pathways.

The figure illustrates how a polluted water body enriched with organic matter, nutrients (nitrogen and phosphorus), and toxicants is transformed into treated water through the synergistic action of a macrophyte–microalgae (AM-MA) consortium. Microalgae, together with their associated microbiome, form biofilms and engage in mutualistic interactions with emergent and submerged macrophytes. Algae photosynthesize, release oxygen, and assimilate dissolved inorganic nutrients; microbiome bacteria degrade organic pollutants and contribute to detoxification; and macrophyte root systems remove excess nitrate and phosphate and sequester heavy metals. These coordinated processes detoxify the water by removing surplus nutrients, pesticides, heavy metals, and other harmful constituents. The resulting consortium biomass acquires a specific “metabolic passport”—i.e., a stress-modulated composition and properties. Under nitrogen limitation and other stressors, microalgae shift their metabolic balance, increasing the lipid fraction relative to proteins and carbohydrates; both algal cells and macrophyte tissues accumulate assimilated elements (N, P, trace metals), and toxic compounds are converted into less bioavailable forms. The right panel outlines valorization pathways for the harvested biomass: production of biofuels, generation of biogas via anaerobic digestion, manufacture of bio-based fertilizers from nutrient-rich biomass, and pyrolysis to biochar for soil improvement. This integrated approach returns recovered nutrients to productive use, yields renewable energy and materials, achieves regulatory water-quality targets, and advances a CBE.

The carbohydrate platform relies on an elevated polysaccharide content in MA biomass (in some regimes, 65% dry weight, DW), making it suitable for bioethanol, biobutanol, and biohydrogen. Cultivation does not compete for arable land or freshwater and is coupled with CO_2_ capture and wastewater treatment [132,133]. Within biorefinery pathways, lipids are directed to transesterification into methyl esters, and carbohydrates to acetone–butanol–ethanol (ABE) fermentation and dark fermentation of hydrogen [134]. Prototypes have demonstrated co-production of biodiesel and bioethanol from psychrophilic Chlamydomonas with total fuel yields in the order of 300 mg·g^−1^ dry biomass under low-temperature cultivation [135]. Biobutanol production has been shown with *Chlorella* grown on municipal wastewater, achieving >6 g·L^−1^ alongside concurrent removal of N and P [136].

Anaerobic digestion remains a core energy-recovery route for AM biomass and residual algal biomass; it proceeds via sequential hydrolysis, acidogenesis, acetogenesis, and methanogenesis to form CH_4_, CO_2_, and H_2_, and it yields a valuable by-product—fertilizer-grade digestate [137,138,139]. For duckweed, enzymatic pretreatment increases the biochemical methane potential compared with untreated biomass [140]. Three-stage schemes combining acidogenic, methanogenic, and electrohydrogenesis blocks show increased overall energy yields and a reduced hydraulic retention time (HRT) [141]. In nature-inspired engineered systems, conversion of AM-MA biomass is accompanied by measurable electricity generation [32].

Thermochemical routes, pyrolysis and hydrothermal carbonization, produce biochar with developed porosity and functional groups; the temperature and residence time govern the yield, pH, surface area, and chemistry [142]. For “dirty” matrices, co-pyrolysis of sewage sludge with algal biomass is effective: adding a small fraction of algal biomass reduces ash content and increases biochar yield [143]. Thermal decomposition ranges for key MA components, dehydration 25–200 °C, carbohydrate/protein decomposition 200–500 °C, lipid decomposition 350–550 °C, and further carbonization 550–800 °C, allow tuning of material properties for sorption and soil applications [144]. Applications extend beyond classical soil amelioration, including the reduction of hematite to metallic iron using algal biochar as a high-temperature reductant under inert atmosphere [145].

Biohydrogen obtained by dark fermentation of algal biomass combines technological simplicity with scalability [146]. The most universal pretreatment for polysaccharide solubilization is dilute-acid hydrolysis, often with thermal assistance; the approach has been demonstrated for *Chlorella*, *Scenedesmus*, and *Dunaliella* [147]. With domestic sewage, high H_2_ yields have been achieved in parallel with high growth productivity and removal of N and P for *Chlorella pyrenoidosa*, *Scenedesmus obliquus*, and *Chlorella sorokiniana* [148]. For dairy wastewater, an effective two-stage platform involves native bacteria and carbohydrate-rich cultures of *Chlorella* and *Clostridium butyricum* with automatic pH control [149].

From a system ecodesign standpoint, a “safety loop” is critical. It includes mandatory analytical screening of biomass for heavy metals and xenobiotics, branching of streams into food-grade versus energy pathways, and when thresholds are exceeded, targeted detoxification and sequestration methods, including membrane and sorption solutions, with traceability of product fractions [79,80]. Under stress-induced lipogenesis, such measures are especially important, as growth of the energy-dense lipid fraction can be accompanied by co-transfer of contaminants into target products [87,150]. Thus, integrating AMs and MAs in engineered ecosystems ensures the required efficiency of water purification and generates biomass suitable for diversified valorization. The biorefinery approach accounts for the feedstock’s “metabolic passport” and selects technological routes in accordance with its chemical profile and contaminant load: from lipid transesterification and carbohydrate fermentation to anaerobic digestion and thermochemical transformations with metal immobilization [118,122,144].

## 4. Challenges and Opportunities of Phyto-Algal Consortia

Implementing CBE principles creates both short- and long-term benefits: reduced consumption of primary resources and waste generation, lower handling costs with funds freed for innovative investment, and, strategically, conservation of limited resources and a lower overall environmental footprint, yielding a “cleaner” environment for future generations [151]. In the CE logic, microalgal biorefineries integrate wastewater treatment with the production of a broad range of bioproducts, seeking to maximize utilization of all biomass fractions and ensure commercial viability [152].

Nature-based treatment systems featuring macrophytes deliver substantial nutrient removal but come with ecological and management risks. Fluctuations in water level, temperature, pH, and salinity induce physiological stress, slowing growth and diminishing pollutant removal. Complex mixtures of nitrogen, phosphorus, heavy metals, pharmaceuticals, and other organic micropollutants complicate transformation pathways and intensify plant inhibition. Risk reduction relies on species-specific selection of tolerant taxa, hydraulic control via hydraulic residence time and buffer volumes, cascade/stepwise configurations, and monitoring of priority pollutant classes with early adjustment of operating regimes before irreversible stress sets in [120,153].

Effectiveness depends not only on the macrophytes themselves. Coupled communities—epiphytes and periphyton, macroinvertebrates and fish—play key roles. Greater habitat complexity and diversity of periphytic algae correlate with lower nutrient concentrations and higher water clarity. Design choices should therefore intentionally enhance biodiversity and trophic cascades, for example, through mosaic vegetation patches and installation of substrates for periphyton [154]. Ecosystem disservices must also be considered. In human-altered water bodies, dominance by a few taxa degrades regulating and cultural ecosystem services. Proper attribution of macrophyte contributions requires comparisons of stressed and reference systems. Highly productive species such as Eichhornia crassipes are often effective at pollutant removal but prone to invasion, clog waterways, deoxygenate water, and displace native biota. To minimize risks, local species and strains should be prioritized, planting material tightly regulated, biomass selectively harvested on schedule, and hydrological or physical containment barriers employed [120,155]. Biomass handling remains central to risk management. Macrophytes accumulate pollutants, irregular harvesting leads to secondary pollution, and inadequate disposal remobilizes toxicants. For feed and food applications, biomass from toxic effluents containing heavy metals, endocrine disruptors, and pharmaceuticals must be excluded even at trace levels [153]. Safer strategies emphasize non-food routes—adsorbents, biochar, biodiesel, bioethanol, biogas, and biohydrogen—and the use of thermal, extraction, and microbial treatments for contaminated biomass [156].

Seasonality strongly affects performance. Winter stagnation and dormancy reduce removal rates and alter hydraulics and thermal regimes. Combining species portfolios that include cold-tolerant forms, stepwise trains, buffer volumes, and integration of photoautotrophy with mixo- or heterotrophic modes in high-productivity species such as duckweed helps smooth seasonal troughs and increase target biomass output. These solutions should be aligned in advance with logistics and processing economics, including proximity of harvesting sites to utilization facilities [157,158].

Heavy metals exhibit duality. Macrophytes including *Pistia, Eichhornia*, *Salvinia, Hydrilla*, and *Lemna* effectively reduce loads in water bodies, but this raises requirements for controlling toxicant migration along the “water–biomass–product–environment” chain. Phytomining routes and genetic strategies to enhance accumulation and tolerance remain promising, yet deployment demands stringent safety assessment [159]. For applications with lower sanitary–hygienic stringency, such as irrigation after polishing, water quality must be certified under accepted standards of agricultural suitability [160]. Greater removal of persistent organic pollutants warrants combined strategies, including pairing macrophytes with growth-promoting rhizosphere microorganisms and integrating chemical and biological pre- and post-processes [156].

Wastewater-grown biomass can contain metals and organic micropollutants; food/feed routes are allowable only after demonstrated compliance with binding limits. At most sites, the realistic outlet is energy/materials rather than food/feed. A pragmatic two-stream principle is recommended. Biomass with minimal contamination may be used, at most, as a biofertilizer or soil amendment, and only after documented compliance with binding contaminant limits and agronomic safety requirements, including pathogen inactivation and leachability control under the applicable fertilizer regulations. Biomass that is more contaminated or has a high moisture content should be processed via thermochemical conversion, hydrothermal liquefaction, pyrolysis, or gasification with verified immobilization of pollutants and rigorous monitoring and management of all by-products; direct land application is not appropriate in this case.

### 4.1. Contemporary Engineering Solutions and Scale-Up Pathways

Reactor choice and CO_2_ management. Microalgae show strong potential for sustainable wastewater treatment and resource recovery. Practical deployment requires targeted screening and adaptation of strains to specific pollutants and variable environmental conditions, since taxa differ in tolerance and in metabolic removal pathways [161,162]. Clear biosafety and chemical-safety rules are also needed, especially when using genetically modified cultures, together with protocols for real effluents [15]. Engineering choices between high-productivity open ponds (HRAP) and closed photobioreactors must account for land requirements, seasonal light dynamics, light distribution in the volume, heat and mass transfer, and CO_2_ availability. Supplying point streams of CO_2_ markedly increases productivity and process robustness [163,164]. Thin-layer cascades (TLCs) are effective for intensification due to their shallow culture depth; stability is supported by pH control and mixing strategies demonstrated for raceway channels and TLCs [8]. The integration of photocatalytic modules into bioelectrochemical systems has emerged as a next-generation strategy to intensify photobioelectrochemical processes. By transducing incident light into catalytic redox activity, such hybrid configurations stimulate the proliferation of photoautotrophic microorganisms and biofilm phototrophs, while simultaneously enabling the degradation of electroactive contaminants, the recovery and generation of energy carriers such as hydrogen, and the mitigation of CO_2_ emissions [165]. In open HRAP, diurnal/seasonal variability necessitates climatic tailoring and careful TEA-driven optimization [166].

Stability and mixed consortia. Robustness to wastewater variability increases with mixed microalgae–bacteria communities, use of local strains and accessible organic substrates, improved light transmission in turbid media, and active management of culture contamination. Biomass grown on wastewater may contain metals and organic micropollutants; food and feed routes are viable only after safety is confirmed. In practice, energy- and materials-oriented pathways (biogas, biodiesel, biochar, fertilizers) are often preferable [167]. Biorefinery logic calls for complementing energy products with high-value compounds (antioxidants, carotenoids, polyunsaturated fatty acids) and for employing hydrothermal and other thermochemical conversions of wet biomass to raise resource efficiency [168]. The trajectory toward biopolymers and bioplastics strengthens circular production–consumption models but requires targeted solutions to overcome techno-economic barriers [169]. Viability should be evaluated via LCA/TEA to identify the dominant impact and cost drivers and to fine-tune reactor configurations, lighting regimes, and product portfolios ahead of scale-up [170].

Harvesting and concentration. Separating dilute microalgal suspensions remains a bottleneck, because the high water content and micrometer-scale cells increase the energy demand for solid–liquid separation. Current practice therefore emphasizes bioflocculation or autoflocculation, natural polymer coagulants (for example, chitosan), growth as immobilized biofilms, and membrane retention; these approaches simplify stream separation and increase the solid content of the harvested biomass [164].

Membrane polishing and spacers. To meet water reuse targets after the biological stage, hybrid treatment trains increasingly incorporate membrane operations such as reverse osmosis (RO), forward osmosis (FO), pressure-retarded osmosis (PRO), and osmotically assisted reverse osmosis (OARO). In spiral-wound modules, channel spacers are performance-critical because their geometry and materials govern local hydrodynamics, mass transfer, concentration polarization, and the propensity for biofouling and mineral scaling. When membrane polishing is coupled with well-optimized, high-rate algal ponds (HRAPs) and plant-wide materials management, systems can deliver reuse-grade effluent with competitive energy and environmental performance. Evidence includes consolidated HRAP design and operational insights that inform polishing needs, comparative life cycle analysis of algal–osmosis membrane trains versus conventional advanced potable reuse that demonstrates favorable energy and greenhouse-gas profiles for the integrated pathway, and pilot-scale flow-reversal RO showing high overall water recovery without loss of permeate quality, which reduces concentrate handling and extends membrane life. Spacers, as structural and hydraulic modifiers, are indispensable for optimizing shear rates, minimizing fouling, and enhancing solute back-transport. Recent advancements in spacer engineering include machine-learning-assisted optimization of geometric patterns and the development of multifunctional coatings with anti-biofouling and anti-scaling properties, which together improve membrane longevity and system performance [171,172,173,174].

Cascaded macrophyte–microalgae systems. Stepwise configurations distribute load and light: macrophytes rapidly shave ammonium and phosphate peaks and partially clarify the water; microalgae then perform polishing and accumulate residual contaminants [113]. The best results in nature-based complexes come from combining HRAP for microalgae with ponds containing floating macrophytes. Managing hydraulic retention time and seasonal “light windows” keeps effluent ammonium and phosphorus low while increasing energy-grade biomass; efficiency gains are associated with longer HRT and with accounting for radiation balance and temperature, which are critical for nitrification, denitrification, and phosphorus accumulation [175]. For agro-industrial effluents, coupling constructed wetlands (CWs) with duckweed and microalgae is promising; harvest engineering is key, as duckweed starch can be converted into low-cost flocculant, closing internal loops and reducing OPEX while increasing yields of processable biomass [176]. Integration with CO_2_ capture, up to supplying flue gases, accelerates growth and carbon fixation by microalgae while macrophytes stabilize nutrient fluxes; benchmarking requires LCA (carbon fixation, N/P recovery, product fate) and TEA [177,178].

CBE alignment (integration, metrics, product portfolios). To align with CBE, systems must return both water and nutrients while maintaining low specific energy use with minimal chemical inputs; this is achieved by optimizing HRAP and deploying engineering solutions for managing shading and contamination [179]. Significant CAPEX/OPEX reductions are possible when real wastewater is used as the nutrient medium, when energy-efficient, thin-layer cascades and raceway/flume channels are selected, and when labor intensity is reduced. Additional benefit arises when the cost of treatment is credited in the project’s overall economics [180]. Strategic development should align with sustainability goals and rest on partnerships among science, industry, and government, accelerating photobiotechnology deployment, safe regulatory frameworks, and expansion of microalgal applications in water, energy, and materials [181].

Embedding macrophyte-based solutions into CBE models implies returning nitrogen, phosphorus, and organic resources while producing bioproducts. This requires institutional support through economic incentives, cross-disciplinary cooperation, and clear stratification of biomass-use trajectories for minimally contaminated biomass versus contaminated biomass. Feasibility should be confirmed by life cycle assessment (LCA) and techno-economic analysis (TEA), with sensitivity analysis and modeling of logistics and processing scales [153,158]. Moving to biopolymers/bioplastics supports circularity but still faces scale and cost hurdles [169].

Scale-up should be gated by site-specific LCA/TEA, as dewatering, CO_2_ supply, seasonality, and co-product choices dominate viability [170]. Near-term priorities: configurations for rural areas without sewerage, management of macrophyte–microbiome interfaces, timely harvesting before winter remobilization/leaching, and breeding/biotech to deliver robust taxa with higher dissolved-pollutant uptake [182].

### 4.2. Barriers to Scale-Up and Enabling Measures

**Figure 2:**
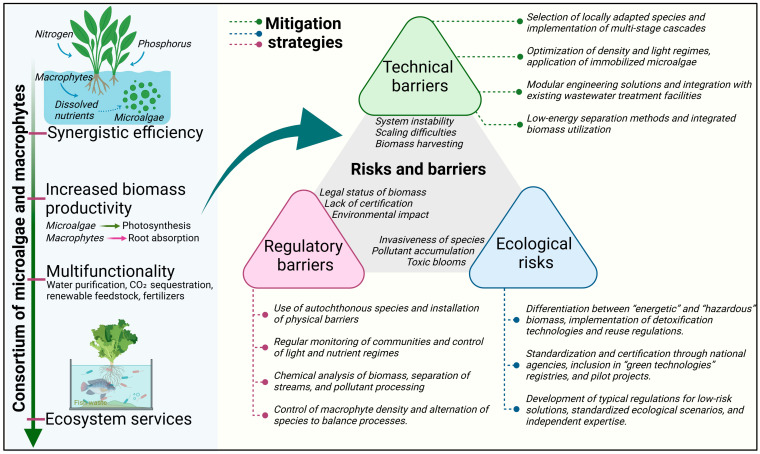
Barriers and strategies for scaling up a hybrid water purification system based on microalgae and macrophytes. Created with BioRender.com (agreement number: AR28NUTNOE). Available online: https://BioRender.com/qwaobzs (accessed on 26 August 2025).

The transition from pilot projects to scalable implementation faces several obstacles. Figure 2 provides a brief overview of barriers and strategies for overcoming them in such hybrid systems. The primary challenges encountered during the implementation and expansion of such hybrid bio-purification systems that combine microalgae and macrophytes are illustrated schematically, and strategies for overcoming these barriers are proposed. These obstacles are conventionally divided into three categories: technical, regulatory, and environmental. Appropriate solutions have been developed for each category.

Technical barriers. Key issues include maintaining a stable consortium performance under variable conditions, scaling laboratory set-ups to industrial units, and efficient harvesting and co-processing of mixed biomass. Mitigation measures comprise selecting local, well-adapted microalgal and plant taxa; optimizing cultivation conditions (biomass density, illumination, nutrient regimes); employing multistage cascade reactors to increase process reliability; immobilizing microalgae (cell attachment to carriers) to facilitate downstream separation; modular plant design with integration into existing treatment facilities; and adoption of low-energy separation methods for joint biomass processing.

Regulatory barriers. Legal and normative challenges include the unclear status of the resulting biomass (waste vs. resource), lack of approved standards and rules governing these biotechnologies and their products, and the need for environmental assessments prior to deployment. Overcoming these barriers requires coherent legal frameworks that define biomass status and permissible uses, certification of derived products (e.g., biofertilizers or biofuels), and regulation of the use of living organisms for water treatment. Interdisciplinary collaboration among lawyers, ecologists, and engineers is essential to co-develop standards that ensure treatment efficacy and environmental safety.

Environmental risks. The treatment system must be environmentally safe. Risks include uncontrolled spread or invasiveness of introduced species, excessive algal blooms with toxin release, and secondary pollution if pollutant-laden biomass (e.g., heavy metals) is improperly handled. Risk-reduction strategies comprise exclusive use of autochthonous species, physical containment or closed systems to prevent organism escape, regular monitoring of community status (microalgal composition, macrophyte development, microbial activity) and water quality to allow early suppression of blooms or toxin accumulation, and safe downstream processing or disposal, which prevents re-release of concentrated contaminants.

In summary, successful transition from experimental setups to full-scale application hinges on a coordinated, interdisciplinary effort. The joint involvement of ecologists, hydrobiologists, engineers, biotechnologists, and regulatory experts is essential at all stages—from system design and species selection to reactor optimization and policy alignment. Prioritizing local biodiversity, implementing well-calibrated cascade systems, and aligning with robust regulatory standards ensures both ecological safety and operational efficiency. Ultimately, such a foundation enables AM-MA consortia to evolve into scalable, circular solutions. When combined with thorough life cycle and techno-economic assessments, thoughtful spacer design in membrane-integrated systems, and flexible valorization routes, these bioengineered platforms can bridge the gap between environmental restoration and the production of renewable bio-based resources [113,120,153,154,155,156,157,158,170,171,172,173,174,175,176,177].

## 5. Conclusions

Phyto-algal consortia are increasingly regarded as a unified engineering platform that links circular wastewater treatment with biomass valorization. In these systems, macrophytes buffer nitrogen and phosphorus loads, stabilize hydraulics and light, and structure the root zone. Microalgae complete effluent polishing, fix CO_2_ into biomass, and supply feedstock suitable for biorefinery. Performance arises from coordinated pathways including uptake and storage of N and P, sorption and co-precipitation on biofilms and root surfaces, redox conversions in rhizo- and periphytic layers, and turbidity reduction, which improves the light field. These mechanisms are implemented through hydraulic cascades, controlled illumination, local aerobic and anaerobic microzones, bio- and autoflocculation, immobilized growth, and membrane retention. Economic viability is strongest when the treatment train is conceived as a biorefinery. Clean biomass streams are routed to fuel and materials products. Fractions that carry impurities undergo thermochemical conversion to biochar and adsorbents with verified immobilization. Complete removal of pharmaceutical micropollutants cannot be assumed in nature-based configurations and may require hybrid stages or dedicated adsorbents.

Looking ahead, we expect gradual but tangible progress across five complementary areas, and several approaches remain precommercial. (i) Genetic and synthetic ecology upgrades of algae and associated microbiomes are promising, although CRISPR-based strategies for stress and xenobiotic tolerance are at laboratory to pilot maturity and will require regulatory review. (ii) Process intensification with light management, such as thin-layer cascades, coupling to point-source CO_2_, and membrane retention, can decouple hydraulics from solids residence, but energy and materials balances should be verified across seasons. (iii) Digital twins with online nutrient and optical sensing and model-predictive control can mitigate seasonal and influent variability, although broader deployment depends on the standardization of sensors and control algorithms. (iv) Integrated low-CAPEX harvesting using biopolymer flocculants, magnetic assistance, and low reagent dewatering can reduce operating inputs, provided choices match downstream conversion needs. (v) Selective valorization with explicit safety gates prevents contaminant recirculation and enforces auditable product quality thresholds. The near-term implementation agenda is clear: screen and adapt local taxa under real effluents and climates, codify biomass traceability and safety, and complete a life cycle assessment and techno-economic analysis that credit avoided treatment costs and carbon capture. With harmonized pilot protocols, water reuse standards, and suitable financing instruments, phyto-algal consortia can advance from demonstrations to modular, bankable water energy systems that meet regulations, enable nutrient circularity, and supply low-carbon products.

## Figures and Tables

**Table 1:** Efficiency and mechanisms of removal of aquatic pollutants by phyto-algal consortia.

Pollutant/Feedstock	Consortium Composition	Removal Efficiency (Approx.)	Key Mechanisms and Notes	Ref.
Municipal wastewater (COD, NH_4_–N, TKN, PO_4_–P)	*Chlorella sorokiniana* (UTEX 1230) + *Lemna minor*	COD—99%; NH_4_–N—90%; TKN—88%; PO_4_–P—91%	Sequential reactors: microalgae grow on raw influent and remove carbon and phosphate; duckweed polishes ammonium/TKN; hetero/mixotrophic uptake, nitrification–denitrification, NH_3_ stripping, Ca/Mg-phosphate precipitation; stable operation with high biomass yields	[111]
Textile wastewater (azo dyes; COD, BOD_5_, TDS)	*Pistia stratiotes*/ *Chlorella vulgaris*	*Pistia*: decolorization 84; COD—61; BOD_5_—71.9; TDS—72. *Chlorella*: decolorization 99 (batch)/74 (scale-up); phytotoxicity ↓ (seed germination 80% vs. 30% untreated)	*Pistia*: adsorption/biosorption on roots and biofilms, enzymatic azo-group reduction; rhizosphere co-biodegradation. *Chlorella*: biosorption + intracellular enzymatic breakdown; photosynthetically induced coagulation/precipitation at elevated pH	[112]
Cheese-making wastewater	*Lemna minor* (PBR-1) + *Chlorella* sp. or *Scenedesmus* sp. (PBR-2)	COD—60%; NO_3_-N—90–100%; PO_4_-P/SO_4_^2−^: partial at RW—30% → complete at TRW—10% after PBR-2	Two-stage phyto-algal system: duckweed removes suspended solids and assimilates N/P; microalgae grow on treated effluent and assimilate residual nutrients/organics; sequential design improves carbohydrate/lipid content in biomass and facilitates harvesting	[113]
Swine wastewater (NH_4_–N, P, pathogens)	*Vetiveria zizanioides* + *Dictyosphaerium* sp.	NH_4_–N ↓ to 5 mg L^−1^ in 13 d (vs. ≥34 days in single-culture controls); P ↓ to 2 mg L^−1^; DO > 10 mg L^−1^; *Escherichia* spp. eliminated	Algal photosynthesis supplies O_2_ and ROS (pathogen inactivation); plant root respiration acidifies medium and reduces NH_3_ toxicity; algae deplete HCO_3_^−^; joint C/N/P uptake accelerates removal; synergistic growth	[114]
Mixed heavy metals + nutrients	*Pistia stratiotes*/*Elodea canadensis* + *Ankistrodesmus* sp., *Chlorella vulgaris*	Cd—89%; Zn—93%; Cu—82%; Pb—90%; BOD_5_—93%; nutrients up to 98%	Biosorption/bioaccumulation by plant roots and algal cells; adhesion of algae to macrophyte roots ↑contact/retention; microbial mineralization and co-precipitation; selective metal uptake (Zn > Cu > Cd > Pb)	[115]
Excess nutrients and heavy metals (Cu, Ni)	*Desmodesmus* sp. + *Ampelodesmos mauritanicus*	N removal: 70% (no metals); 59% (with Ni); <7% (with Cu or Cu/Ni mix). P removal: 90% (no metals); 36–39% (with Ni or Cu); 13% (Ni/Cu mix). Metals: Cu—74%; Ni—85% (single); Cu—59% (mix); Ni in mix ≪ 85%	Rapid adsorption on algal cell walls—intracellular accumulation; nutrient uptake by algae and plant roots; competitive binding favors Cu over Ni; root-zone phytoremediation complements algal removal	[116]
Aquaculture wastewater (NO_3_–N, NH_4_–N, TN, TP, COD)	*Chlorella* sp. + *Spirodela polyrhiza*	NO_3_-N—91%; NH_4_-N—99%; TP—100%; TN—92%; COD—95%	Direct N/P uptake by microalgae and duckweed; photosynthetic oxygenation enhances aerobic mineralization; microbial nitrification–denitrification; phosphate sorption/co-precipitation; functionally diverse consortium yields feed/bioenergy-ready biomass	[117]

Abbreviations: COD—chemical oxygen demand; BOD_5_—5-day biochemical oxygen demand; TDS—total dissolved solids; ↓—decrease relative to the control/baseline condition; TKN—total Kjeldahl nitrogen; TN—total nitrogen; TP—total phosphorus; DO—dissolved oxygen; ROS—reactive oxygen species; HCO_3_^−^—bicarbonate; PBR—photobioreactor (PBR-1/PBR-2 = stage 1/2); RW-x%—raw wastewater at x% dilution (feed to PBR-1); TRW-x%—treated raw wastewater from PBR-1 fed to PBR-2 at x%.

## Data Availability

Data available on request from the authors.

## Abbreviations

The following abbreviations are used in this manuscript:

CBE HGT	circular bioeconomics horizontal gene transfer
HRAP	high-rate algal pond
HRT	hydraulic retention time
LCA	life cycle assessment
MP	metabolic passport (of biomass)
MA	microalgae
PBR	photobioreactor
SDGs	United Nations Sustainable Development Goals
SOD	superoxide dismutase
MDA	malondialdehyde
TCA	tricarboxylic acid cycle
TEA	techno-economic analysis
